# Comparison of Diagnostic Efficacy of Contrast-Enhanced Ultrasound, Acoustic Radiation Force Impulse Imaging, and Their Combined Use in Differentiating Focal Solid Thyroid Nodules

**DOI:** 10.1371/journal.pone.0090674

**Published:** 2014-03-03

**Authors:** Jin Deng, Ping Zhou, Shuang-ming Tian, Lu Zhang, Jia-le Li, Ying Qian

**Affiliations:** Department of Ultrasound, the Third Xiangya Hospital, Central South University, Changsha, Hunan, China; Bascom Palmer Eye Institute, University of Miami School of Medicine, United States of America

## Abstract

**Background:**

A key limitation of conventional ultrasound (US) includes poor differentiation of benign from malignant thyroid nodules. Contrast-enhanced US (CEUS) and acoustic radiation force impulse (ARFI) could provide better characterization of focal thyroid nodules; however, no studies have compared their efficacies.

**Objective:**

To evaluate the diagnostic efficacy of conventional US,CEUS, ARFI, and their combined use in differentiating focal solid thyroid nodules.

**Methods:**

One-hundred-forty-six Chinese patients with 175 thyroid nodules (119 benign and 56 malignant) were prospectively enrolled. Each patients underwent conventional US, CEUS and ARFI, respectively. The diagnostic performance of the conventional US, CEUS, ARFI, combined use of either CEUS or ARFI and combined use of the three modalities were assessed and compared using Pathological diagnosis (histological/cytological) as the reference method.

**Results:**

There were no significant differences between individual groups (CEUS vs US, *P = 0.279*, ARFI vs US, *P = 0.372*, CEUS vs ARFI, *P = 0.849*), combined use of US and CEUS or combined use of US and ARFI yielded significant difference compared to US. (combination of US & CEUS vs US, *P = 0.021*; combination of US & ARFI vs US, *P = 0.036*). The combination of three modalities significantly improved the diagnostic accuracy compared with either combination of conventional US and CEUS or combination of conventional US and ARFI (*P* = 0.045 and *P*  = 0.027, respectively).

**Conclusions:**

CEUS and ARFI can be used as an additional tool in the diagnostic work up of thyroid nodules. The combination of CEUS with ARFI can significantly improve the diagnostic accuracy.

## Introduction

The incidence of thyroid nodules has gradually increased in the recent years, and about 5% to 15% of them are malignant nodules [1]. With the development of high-frequency ultrasound (US) imaging techniques, the detection rate of thyroid diseases has greatly been improved [2]. Though conventional US has become the preferred imaging method for diagnosing thyroid diseases, its key limitation includes poor differentiation of benign from malignant nodules. Fine needle aspiration (FNA) is widely adopted by clinicians as a simple, minimally invasive way of diagnosing thyroid nodules with sensitivity and specificity ranged from 65–98% and 72–100%, respectively [3]–[7]. However, this technique may have false positive or negative outcomes [8], and about 10% to 20% of thyroid nodules could not be diagnosed [9]. Hence, new imaging modalities with efficient differential diagnosis of benign and malignant thyroid nodules are needed.

Contrast-enhanced ultrasound (CEUS) is a relatively new modality, which allows studying dynamic enhancement patterns of focal thyroid nodules in real time and thus provides much better characterization of focal thyroid nodules than conventional US. Several studies have shown that CEUS has the ability to efficiently characterize focal thyroid nodules [10]–[12].

Acoustic radiation force impulse (ARFI) is another novel modality that could quantitatively evaluate the tissue stiffness. During ARFI imaging, tissue in the region of interest (ROI) is mechanically excited using short-duration acoustic pulses to generate small (1 µm to 20 µm) localized tissue displacements, which result in shear-wave propagation away from the region of excitation. The quantitative implementation of ARFI is termed as virtual touch tissue quantification (VTQ), which gives an objective numerical evaluation of the tissue stiffness. The speed of shear wave propagation increases with the increase in stiffness of tissue.

A number of studies have reported the diagnostic efficacy of CEUS and ARFI individually. However, to our knowledge, no studies have compared CEUS and ARFI in differentiating benign and malignant thyroid nodules. Hence, the present study was aimed to assess the diagnostic efficacy of CEUS, ARFI, and combined use of both modalities in differentiating benign from malignant thyroid nodules.

## Materials and Methods

### Study design and setting

The present prospective study was conducted at the Third Xiangya Hospital of Central South University, changsha, China between March 2011 and March 2013. The diagnostic efficacy of conventional US, CEUS, ARFI, combined use of conventional US with CEUS, combined use of conventional US with ARFI and combined use of the three modalities were assessed and compared using histological/cytological as the reference. The study protocol and informed consent form were reviewed and approved by the hospital's Ethics Committee, and all subjects provided written informed consent before study enrollment.

### Subjects and selection criteria

Of 161 consecutive patients with thyroid nodules 146 patients (with 175 nodules) were enrolled in the study. The following were the inclusion criteria for the study: 1) patients with histopathology-confirmed (surgery or US-guided biopsy) thyroid nodules (benign or malignant); 2) aged >18 years; and 3) willingness to provide written informed consent. Patients with following criteria were excluded from the study: 1) no cytology by FNAB or histology by surgery of the thyroid nodule within the study period, indeterminate cytology by FNAB without repeated FNAB, and suspicious or malignant cytology by FNAB without thyroid operation within the study period. 2) nodule diameter <5 mm (size of sample ROI for ARFI is 6 mm×5 mm); 3) mixed cystic nodules or almost cystic (<25% solid); 4) huge nodules without surrounding normal thyroid tissue; 5) nodules with immeasurable VTQ value (nodules with onscreen value of X.XX m/s); 6) pregnant women; and 7) aged <18 years. A total of 15 patients were excluded from the study based on the above criteria, and the study consisted of 146 patients in total.

### Demographics and clinical features

Of the 146 patients, 42 were males and 104 were females. The mean age was 46.3±12.5 years (range: 18 to 69 years). The maximum diameters of the lesions ranged from 6 to 45 mm(mean size, 18.2 mm±8.60 mm). There were 175 nodules identified in the study population with 119 benign nodules (103 nodular goiters, 14 thyroid adenomas, and 2 with chronic thyroiditis) and 56 malignant nodules (54 papillary carcinomas and 2 follicular carcinomas). 55 patients had single nodule and 91 patients had multiple nodules in each. For the patients with multiple nodules, the nodules that were highly suspicious of malignancy or the largest one were selected for analysis. Diagnoses of all nodules were based on histopathology/cytology confirmation (surgery or biopsy). The final diagnosis of nodules was obtained using histopathology/cytology examination after surgical resection (benign: 73 lesions, malignant: 49 lesions) and US-guided biopsy (benign: 44 lesions and suspicious cytology from 4 nodules, papillary carcinoma in 5 nodules). All suspicious and malignant nodules on cytology were referred to surgery.

### Equipments and measurements

Siemens Acuson S2000 US machine (Siemens, Mountain View, CA, USA) and CEUS, and ARFI imaging technology were used in this study. A linear array transducer (9L4, Siemens Medical Solutions, Mountain View, CA, USA) with a center frequency of 7.5 MHz (range: 5.0 MHz to 14.0 MHz) was equipped for all conventional US, CEUS, and ARFI examinations. The contrast agent used in the present study was SonoVue (Bracco, Milan, Italy), which contains sulphur hexafluoride microbubbles.

#### Conventional US

Thyroid nodules were evaluated for size, volume, Shape, margin, echogenicity presence/absence of halo-sign, presence/absence of microcalcification,and/or macrocalcification. After B-mode-ultrasound, color Doppler US were performed.

#### ARFI

After conventional US examination, the ARFI scanning were performed. Patients who underwent ARFI examinations were instructed to hold their breath, and the probe was completely placed on the body surface with light pressure on the thyroid. The VTQ values (SWV, m/s) of the nodule were obtained by placing the fixed-size ROI (6×5 mm) to the nodules, and the calcified and liquefaction necrosis portions of the nodule are avoided. After that, the ROI was moved to the surrounding thyroid tissue at the same depth, and the procedure was repeated. The SWV values from the nodules and surrounding thyroid tissue were calculated as the median value of five samplings at the same location within the tumor and surrounding thyroid tissue. The speed of shear wave propagation increased with the increase in stiffness of tissue. Thus it provided numerical measurements that could give quantitative information about tissue elasticity properties.

#### CEUS

A low frame rate (10 Hz) and low mechanical index (MI = 0.1) were used for CEUS. Since bubble disruption was strictly related to depth and focalization of a US beam, focus was always placed deeper than the nodule being examined in order to minimize microbubble disruption. Once the ROI was set, US parameters remained unchanged in each patient. Using a 20-gauge peripheral intravenous cannula, SonoVue was injected intravenously as a bolus at a 2.4 mL dose, followed by 5 mL of normal saline flush. Meantime, the timer on the US machine was started, and the imaging plane was kept as stable as possible. Each contrast imaging acquisition lasted at least 3 minutes after bolus injection and was digitally stored as raw data on a personal computer-based workstation connected to the US unit through a standard Ethernet link.

All examinations were performed by an experienced US physician in order to exclude the bias from different operators. The US imaging data were independently analyzed by two other off-site investigators, who had not performed the CEUS and ARFI examinations and were blind to surgical, histopathological, and other imaging findings. When they did not agree on the evaluation results of some of the diagnostic indices, the nodules were evaluated by another experienced investigator. Each investigator clarified the reasons for making the diagnoses, and a consensus was reached in cases of discrepancies.

### Statistics analysis

Statistical analyses were performed using SPSS software version 19.0 (make). Continuous variables were summarized as means ± SD or median (minimum–maximum). Distribution of variables was analyzed using Kolmogrov-Smirnov test (K-S test). SWV values between benign and malignant nodules were analyzed by Mann-Whitney U non-parameter test. The sensitivity, specificity, positive predictive values (PPV), negative predictive values (NPV), and the diagnostic accuracy of CEUS and ARFI for thyroid nodules were calculated comparing the findings with histological reports and using conventional US as the control. Comparisons of the diagnostic accuracy were performed by χ^2^ test. *P*<0.05 was considered to indicate a significant difference.

## Results

### Conventional US

All patients initially underwent conventional US, and the assessed diagnostic parameters were considered as control. The sensitivity, specificity, and accuracy of conventional US included the following: 71.4%, 81.5%, and 78.3%, respectively.

### CEUS

The 119 benign thyroid nodules showed four enhancement patterns: ring enhancement in 39 nodules (32.8%), isoenhancement in 39 (32.8%), hyperenhancement in 23 (19.8%), and hypoenhancement in 18 nodules (15.1%). The 56 malignant thyroid nodules showed four patterns: isoenhancement in 8 nodules (14.3%), hyperenhancement in 2 (5.6%), and hypoenhancement in 46 (82.1%). No ring enhancement was observed among the malignant thyroid nodules.

Based on the impression that hypoenhancement was considered for malignancy, the sensitivity, specificity, accuracy, PPV, and NPV were assessed to be 82.1%, 84.9%, 84.0%, 71.9%, and 91.0%, respectively.

### VTQ of ARFI

**Figure 1:**
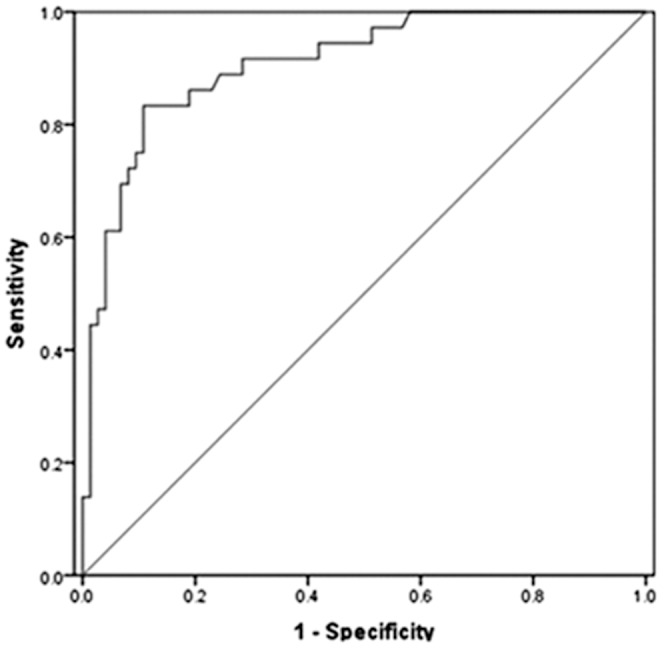
ROC analysis was performed to predict the diagnostic accuracy of malignant nodules on SWV value.

The SWV median of 119 benign thyroid nodules was 2.09 m/s (mean 2.06±0.63 m/s), SWV median of 56 malignant thyroid nodules was 3.04 m/s (mean 3.22±0.92 m/s), and SWV median of 175 surrounding normal thyroid parenchyma was 2.02 m/s (mean 2.02±0.41 m/s). The SWV value of malignant nodules was significantly higher than that of benign nodules (*P* = 0.000). The SWV value of benign nodules was not significantly higher than that of the surrounding normal thyroid parenchyma (*P* = 0.359). ROC curve analyses showed that the AUROC for the SWV was 0.88 (95% CI: 0.83, 0.93) (Figure 1). The sensitivity, specificity, accuracy, PPV, and NPV were reported to be 80.4%, 84.0%, 82.9%, 70.3%, and 90.0% respectively, when the SWV cutoff point was 2.59 m/s.

### Comparison of conventional US, CEUS and ARFI

The final pathological diagnosis was used as a standard for the comparison of conventional US, CEUS, ARFI, and combination of different modalities. The diagnostic performance of different modalities for discrimination between benign and malignant thyroid lesions was shown in table 1.There were no significant differences between individual groups (CEUS vs control, *P* = 0.279; ARFI vs control, *P = 0.372*, CEUS vs ARFI, *P = 0.849*) with respect to sensitivity, specificity, and accuracy. Combined use of US and CEUS or combined use of US and ARFI yielded significant difference compared to control. (combination of US & CEUS vs control, *P = 0.021*; combination of US & ARFI vs control *P = 0.036*).Combined use of three modalities had a higher sensitivity, specificity, accuracy, PPV and NPV of 96.4%, 96.6%, 96.6%, 93.1% and 98.3%, respectively; and it yielded significant difference compared to either combination of conventional US and CEUS or combination of conventional US and ARFI (*P* = 0.045 and *P* = 0.027, respectively).

**Table 1:** **Diagnostic performance of different modalities for discrimination between benign and malignant thyroid lesions.**

methods	Sensitivity (%)	Specificity (%)	Accuracy (%)	PPV (%)	NPV (%)
Conventional US	71.4	81.5	78.3*+	64.5	85.8
CEUS	82.1	84.9	84.0	71.9	91.0
ARFI	80.4	84.0	82.9	70.3	90.0
Combination of US & CEUS	87.5	91.5	90.3#	83.4	94.0
Combination of US & CEUS	85.7	89.9	89.1#	81.3	93.1
Combination of three modalities	96.4	96.6	96.6	93.1	98.3

PPV, positive predictive value; NPV, negative predictive value.

#Significantly different from the combination of three modalities (*P*<0.05).

*Significantly different from the combination of conventional US and CEUS (*P*<0.05).

+Significantly different from the combination of conventional US and ARFI (*P*<0.05).

**Figure 2:**
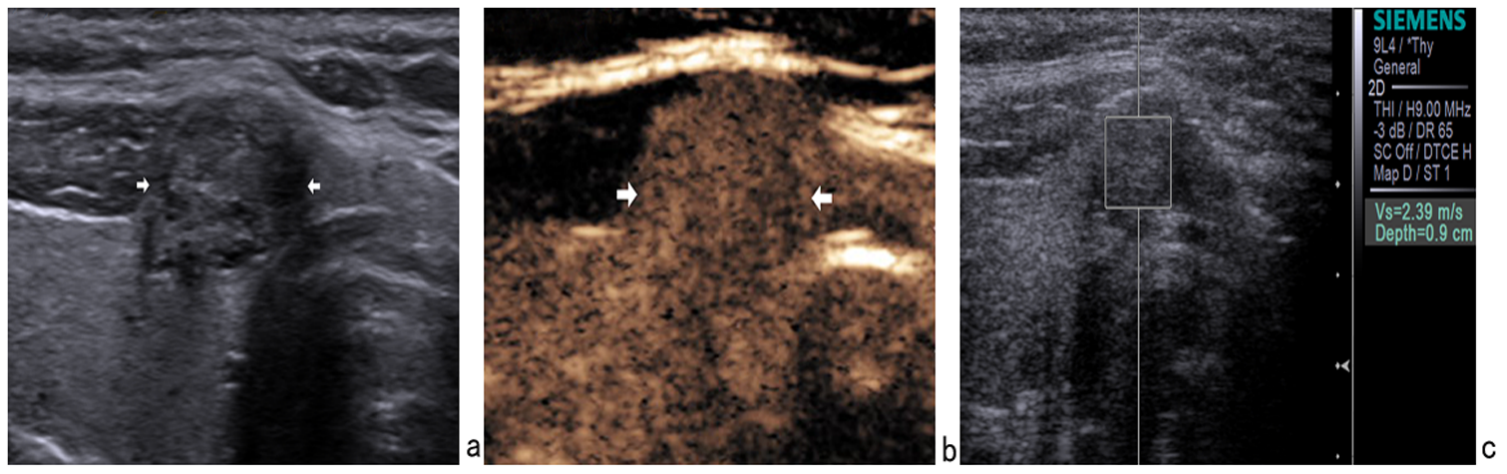
In a 38-year-old female with a 11×8 mm nodule on the inferior segment of the right thyroid lobe, surgical pathology showed a papillary thyroid carcinoma. (a) Gray-scale US showed a hypoechoic nodule with well-defined margin. (b) CEUS showed isoenhancement. (c) The SWV value was 2.39 m/s when the region of interest was placed within the nodule. It was misdiagnosed as benign nodule by conventional US, CEUS and ARFI.

**Figure 3:**
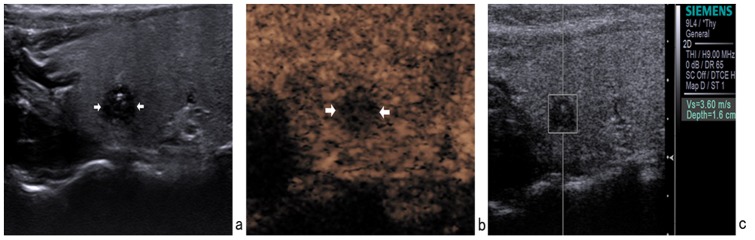
In a 29-year-old female with a 6×5 mm nodule on the right thyroid lobe, surgical pathology showed a nodular goiter. (a) Gray-scale US showed a hypoechoic nodule with ill-defined margin and calcifications. (b) CEUS showed hypoenhancement. (c) The SWV value was 3.60 m/s when the region of interest was placed within the nodule. It was misdiagnosed as malignant nodule by conventional US, CEUS, and ARFI.

**Figure 4:**
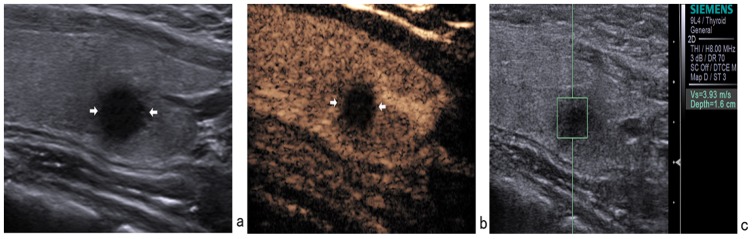
In a 42-year-old female with a 9×8 mm nodule on the left thyroid lobe, surgical pathology showed a subacute thyroiditis. (a) Gray-scale US showed a hypoechoic nodule with ill-defined margin. (b) CEUS showed hypoenhancement. (c) The SWV value was 3.93 m/s when the region of interest was placed within the nodule. It was misdiagnosed as malignant nodule by conventional US, CEUS, and ARFI.

The combined use of three modalities had a concordant result in 169/175 lesions (96.6%) (54 were suspicious of malignancy and 115 were probably benign). Of these 169 lesions, 2 papillary carcinomas were misclassified as benign by combined use of three modalities (Figure 2). Likewise, 1 nodular goiter (Figure 3) and 1 chronic thyroiditis (Figure 4) were misclassified as malignant by combined use of three modalities.

## Discussion

The present prospective study involved the comparison of diagnostic efficacy of CEUS and ARFI in differentiating benign from malignant thyroid nodules in Chinese patients. The findings by conventional US served as the control, while final histopathological diagnosis served as the standard for comparison. As of date, no studies have compared the efficacy of CEUS and ARFI with respect to differential diagnosis. Since the current clinical practice lack evidences on imaging techniques, the study results would significantly contribute to the improvement of diagnosis of thyroid nodules.

CEUS, a new diagnostic modality, was reported to show potentials in differential diagnosis of benign and malignant thyroid nodules. Zhang B et al [11] found that ring enhancement correlated highly with a benign diagnosis of thyroid nodule (sensitivity: 83.0%, specificity: 94.1%, and accuracy: 88.5%); and heterogeneous enhancement correlated highly with a malignant diagnosis (sensitivity: 88.2%, specificity: 92.5%, and accuracy: 90.4%). Nemec et al [12] found that when CEUS was applied after SonoVue(R) administration, there was a sensitivity of 76.9%, specificity of 84.8%, and diagnostic accuracy of 82.6%. In the present study, a sensitivity of 82.1%, specificity of 84.9%, diagnostic accuracy of 84.0%, PPV of 71.9% and NPV of 91.0% were achieved when thyroid nodules were diagnosed by CEUS. These results were consistent with those of Zhang B [11] and Nemec [12]; and these results indicated the feasibility of CEUS in differential diagnosis of benign and malignant thyroid nodules. In the present study, the majority of 54 malignant nodules were papillary thyroid carcinoma and 46 of 56 (82.1%) showed hypoenhancement. This could be attributed to the lack of blood supply in papillary thyroid carcinoma [13], which might be due to following aspects: 1) papillary thyroid carcinoma grows as invading adjacent tissues, with complex neovascularization inside the tumor [14]; and as tumor growth outweighs neovascularization, necrosis happens within the tumor; 2) the possible cancer embolus may lead to stenosis, which could even block blood vessels; 3) intensive interstitial fibrosis may often come up in papillary thyroid carcinoma [13], the increase in vascularity is usually related to the cellular proliferation in a neoplastic condition, while fibrosis could decrease the density of the vascularity inside the nodules [13], [15]; and 4) thyroid peripheral calcification are psammoma bodies which are usually located on the papillary carcinomas. The 119 benign nodules showed ring enhancement, isoenhancement or hyperenhancement, implying benign thyroid nodules are mostly rich in blood supply, These results were consistent with those of Hee Jung Moon[13]. Multiple imaging patterns of benign nodules results from the variation of blood supply in different stage of the nodules [16].

ARFI has been introduced to objectively evaluate hardness and to improve the diagnostic performance of gray-scale US examination in differential diagnosis of thyroid nodules. Many previous studies have proved that ARFI is useful in differentiating malignant from benign nodules [17]–[21]. Jiying Gu et al [19] reported that the sensitivity, specificity, accuracy, PPV, and NPV for VTQ (cutoff value = 2.55 m/s) were 86.4%, 93.4%, 91.8%, 79.2%, and 95.9%, respectively. Yi-Feng Zhang et al [20] reported that the sensitivity, specificity, accuracy, PPV, and NPV for VTQ (cutoff value = 2.87 m/s) were 75%, 82.2%, 80.3%, 65.1%, and 90.5%, respectively. In the present study, the sensitivity, specificity, accuracy, PPV, and NPV were 80.4%, 84.0%, 82.9%, 70.3%, and 90.0%, respectively. The best SWV cutoff point was 2.59 m/s in the present study, which was consistent with reported range of 2.42 to 3.30 m/s in previous studies [18]–[21].

Several previous studies have reported that the diagnostic performance of CEUS and ARFI in differentiating benign from malignant thyroid nodules. However, this is the first study to compare the efficacy of CEUS and ARFI with respect to the differential diagnosis of thyroid nodules. Results showed that no significant difference was observed in the diagnostic performance among CEUS and ARFI; and comparison with either combination of conventional US and CEUS or combination of conventional US and ARFI, combined use of three modalities had a better accuracy than each modality alone. The overall concordance between the combined use of three modalities was 96.6% (169 of 175 lesions); and of the 169 lesions, two papillary carcinomas were misclassified as benign owing to the focal cancerous nodules whose tissue structure was nearly same as that of normal tissue. One nodular goiter and 1 chronic thyroiditis were misclassified as malignant. These might be correlated with extensive fibrosis and papillose cells with microcalcifications in the interstitium, which could increase stiffness of the nodules and hypovascularity that could produce an enhancement pattern similar to that of malignant tumor.

The current study had some limitations. First, only a few pathologic types of benign and malignant nodules were studied, and the sample size was too small to stand for all kinds of nodules. Second, cystic and solid nodules with diameters <5 mm were excluded from this study because ARFI for them could not give useful information. Third, 54 of 56 malignancies were papillary thyroid carcinomas. There were only 2case of follicular carcinomas. Hence, further studies with more sample size and varied tumor types are required to validate the study results and further analyze and compare the efficacy of CEUS and ARFI in follicular neoplasm.

In conclusion, CEUS and ARFI have been shown potent and equal value in the characterization of thyroid nodules between the benign and malignant nodules. The combined use of conventional CEUS and ARFI can significantly improve the diagnostic accuracy.
